# Can multi-cropping affect soil microbial stoichiometry and functional diversity, decreasing potential soil-borne pathogens? A study on European organic vegetable cropping systems

**DOI:** 10.3389/fpls.2022.952910

**Published:** 2022-09-27

**Authors:** Alessandra Trinchera, Melania Migliore, Dylan Warren Raffa, Sarah Ommeslag, Jane Debode, Sindhuja Shanmugam, Sandra Dane, Joran Babry, Pirjo Kivijarvi, Hanne Lakkemborg Kristensen, Liga Lepse, Tapio Salo, Gabriele Campanelli, Koen Willekens

**Affiliations:** ^1^Council for Agricultural Research and Economics-Research Centre for Agriculture and Environment, Rome, Italy; ^2^Plant Sciences Unit, Flanders Research Institute for Agriculture, Fisheries and Food (ILVO), Merelbeke, Belgium; ^3^Department of Food Science, Aarhus University, Aarhus, Denmark; ^4^Latvian Institute of Horticulture, LatHort, Dobeles Novads, Latvia; ^5^INAGRO, Roeselare, Belgium; ^6^LUKE (FI) Natural Resources Institute Finland, Helsinki, Finland; ^7^Council for Agricultural Research and Economics-Research Centre for Vegetable and Ornamental Crops, Monsampolo del Tronto, Italy

**Keywords:** intercropping, rhizosphere microbial community, root mycorrhization, nutrients, organic vegetables

## Abstract

Crop diversification in spatial and temporal patterns can optimize the synchronization of nutrients plant demand and availability in soils, as plant diversity and soil microbial communities are the main drivers of biogeochemical C and nutrient cycling. The introduction of multi-cropping in organic vegetable production can represent a key strategy to ensure efficient complementation mediated by soil microbiota, including beneficial mycorrhizal fungi. This study shows the effect of the introduction of multi-cropping in five European organic vegetable systems (South-West: Italy; North-West: Denmark and Belgium; North-East: Finland and Latvia) on: (i) soil physicochemical parameters; (ii) soil microbial biomass stoichiometry; (iii) crop root mycorrhization; (iv) bacterial and fungal diversity and composition in crop rhizosphere; (v) relative abundance of selected fungal pathogens species. In each site, three cropping systems were considered: (1) crop 1—monocropping; (2) crop 2—monocropping; (3) crop 1—crop 2—intercropping or strip cropping. Results showed that, just before harvest, multi-cropping can increase soil microbial biomass amount and shape microbial community toward a predominance of some bacteria or fungi phyla, in the function of soil nutrient availability. We mainly observed a selection effect of crop type on rhizosphere microbiota. Particularly, *Bacteroidetes* and *Mortierellomycota* relative abundances in rhizosphere soil resulted in suitable ecological indicators of the positive effect of plant diversity in field, the first ones attesting an improved C and P cycles in soil and the second ones a reduced soil pathogens' pressure. Plant diversity also increased the root mycorrhizal colonization between the intercropped crops that, when properly selected, can also reduce the relative abundance of potential soil-borne pathogens, with a positive effect on crop productivity in long term.

## Introduction

The concept of soil health is unavoidably connected to its multifunctionality and is strongly dependent on soil biodiversity. The recently changed and still evolving environmental conditions, call for management practices able to increase biodiversity and the functional redundancy of soil biological communities to ensure adequate ecosystem resilience (Griffiths et al., 2000).

The introduction of agroecological practices based on crop diversification represents one of the key strategies to ensure efficient complementation and exploitation of energy and nutrients by the soil biota, so to increase the ecosystem resilience (Tsiafouli et al., 2015). Among the alternative strategies to increase crop diversification, intercropping (IC) and strip cropping (SC), where multiple crops are contemporarily grown, can supply several ecosystem services, including nutrient cycles, crop production, control of pests, and diseases (Theunissen, 1997; Li et al., 2007, 2014; Brooker et al., 2015; Ciaccia et al., 2015; Campanelli et al., 2019; Fan et al., 2020). Particularly, crop diversification in spatial and temporal patterns can optimize the synchronization of plant nutrient demand and availability and contribute to increasing the diversity of plant and soil microbial communities, which are among the main drivers of biogeochemical C and nutrient cycle (Crews and Peoples, 2005; Prommer et al., 2019). Understanding the composition, traits, and functions of soil organisms, as well as their ecological interactions, is imperative to understand which agricultural practices can maintain plant biodiversity, soil health, and productivity (van der Heijden et al., 1998; Francaviglia et al., 2004; Zhang J. et al., 2020). Recently, it was found that crop diversification and soil disturbance strongly impact on microbiome functional diversity, as plant residues play a substantial role in defining the assortment of microbial species (Figueiredo Santos and Olivares, 2021; Orrú et al., 2021). The fungi:bacteria ratio in soil microbial community depends on environmental changes and its impact on soil functioning is a key element in the assessment of microbiome functional diversity (Strickland and Rousk, 2010). Suitable parameters and methods to study the functional diversity of soil communities include the fungi:bacteria ratio, the assessment of soil microbial C:N:P stoichiometry, the geno- and phenotype profiling of soil microbial communities, and functional, metagenomic approaches.

Elemental composition and stoichiometry of microorganisms usually stem from indirect analysis of the whole community, although in grassland ecosystems C:P and C:N ratios were found significantly higher in fungi, such as *Ascomycota* and *Basidiomycota*, than in bacteria, such as *Proteobacteria, Bacteroidetes*, and *Actinobacteria* (Mouginot et al., 2014). However, the observed mean stoichiometric ratios fell within the overall distributions reported in Cleveland and Liptzin (2007) and overlap with some recorded ratios for the microbial biomass of the whole communities. The microbial stoichiometry supplies information about organic matter decomposition, patterns of nutrient limitation, and links between fluxes of C, N, and P (Philippot et al., 2013; Mouginot et al., 2014). Anyway, soil nutrient stoichiometry was recognized as the main predictor of bacterial and fungal diversity and composition even at a regional scale, being mainly driven by variation in soil C, N, and P resources, as affected by system management (Delgado-Baquerizo et al., 2017; Bragazza et al., 2021).

When evaluating the effect of agricultural practices on soil microbial stoichiometry, the total soil organic carbon (TOC) content and nutrient availability should be considered. The quantification of mineral N (Keeney and Bremner, 1966) and the estimation of soluble organic C (HWC) and easily available P (HWP) in hot water represent simple methods to determine the C and available P (P_av_) in soils. Hot water extraction leads to the decomposition of organic compounds due to the high temperature (Vanden Nest et al., 2014). HWC and HWP pools are often used as proxies for microbial C (Sparling et al., 1998; Ghani et al., 2003), due to the ability of soil microbiome to stabilize soil organic matter (SOM) within the soil C and nutrient dynamics.

In a review by Strickland and Rousk (2010) about the bacterial or fungal dominance in soils in response to environmental changes, the distribution of C:N ratio of bacteria, saprotrophic fungi, and mycorrhizal fungi was evaluated. The authors found that the dominance of bacteria communities corresponds to a microbial C/N ratio two times lower (between 5 and 6) than under saprotrophic and mycorrhizal fungi dominance (between 9 and 17). Recent results on sugarcane–soybean IC showed that microbial C (C_mic_) and microbial N (N_mic_) increased under IC when compared to monoculture (MC), with a significant increase in microbial C:N ratio especially in intercropped soybean (Lian et al., 2019).

Coupling soil microbiome stoichiometry data with comprehension of soil microbial diversity and functioning give the chance to understand how soil microbiome may be driven by both the introduced crop diversity and soil nutrient availability. In recent years, the massive use of Next Generation Sequencing (NGS) technologies, a high-throughput method to investigate sequences of nucleotides within DNA/RNA molecules (Metzker, 2010; Klindworth et al., 2013), became a common tool in agroecological studies. This method allows us to focus on multiple species (i.e., bacterial and fungi communities) and their related functionality in soil (Moscatelli et al., 2018), for example, nutrient availability, stress resistance, or plant diseases (Sirangelo and Calabrò, 2020). The use of NGS to investigate agricultural soils, where crop diversification and no tillage, was applied for 10 years showed that some fungal species worked as indicators of soil disturbance in intensively tilled soils, while others seemed to be mostly associated with chemical characteristics of plant residues accumulated on soil surface (Ashworth et al., 2017; Orrú et al., 2021).

In general terms, a high microbial diversity is expected in highly diversified cropping systems. In fact, a positive effect of legume–cereal crop rotations on beneficial fungi (e.g., mycorrhizae) was found under Mediterranean conditions, being strongly correlated to SOC, and to occurrence and β-diversity of arbuscular mycorrhiza fungi genera (Pellegrino et al., 2020). In addition, crop diversification (e.g., IC, living mulch, and cover crops) seems to promote the beneficial plant–fungi symbiotic association (Trinchera et al., 2019, 2021).

In organic vegetable farming systems, the farmers often introduce diversification strategies as a mean to control the development of soil-borne plant pathogens. Although some papers have focused on the effects of crop diversification on soil microbiome (e.g., Li and Wu, 2018; Orrú et al., 2021), very few studies investigated how multi-cropping systems or crop rotation affect fungal communities, particularly on those with potential pathogenic activity in soils. Several pathogens were found to be reduced in a cereal–legume multi-cropping system, where wheat and maize were intercropped with faba bean (Wang et al., 2021). Similarly, in greenhouse vegetable production, a tomato–celery rotation was able to trigger a shift of fungal diversity toward less pathogenic populations (Lyu et al., 2020).

The SureVeg project “*Strip-cropping and recycling of waste for biodiverse and resoURce-Efficient intensive VEGetable production*” (CoreOrganic Cofund 2016-2021) aimed at developing and implementing new diversified, resource-efficient, and intensive organic vegetable systems in Europe. In this framework, we focused on the effect of multi-cropping on a set of biochemical and microbial indicators in different European pedoclimatic conditions, to disclose how crop diversification affects diversity and functionality of the soil microbial community and related ecosystem services. We hypothesized that increased plant diversity in multi-cropping can modify the soil microbial stoichiometry, shape the composition and diversity of bacteria and fungi communities, thus improving soil C-N-P cycles mediated by soil microbiota, promoting beneficial plant–fungal symbioses, and containing fungal diseases which afflicted horticultural crop production in those systems (e.g., *Fusarium, Olpidum Brassicae*).

In each experimental site, two crops, representative of the local farming systems, were intercropped row-by-row (IC) or bed-by-bed (SC), compared to MC. This study reports the results obtained on: (i) soil physicochemical parameters; (ii) soil microbial biomass stoichiometry, (iii) crop root mycorrhization; (iv) bacterial and fungal diversity in crop rhizosphere; and (v) relative abundance of selected potential pathogenic fungal species.

## Materials and methods

### Site and experimental design

The SureVeg field trials were established in 2018 in five experimental sites in Europe, representative of different pedoclimatic conditions:

- South-West EU: CREA (Italy, Mediterranean North region)
- North-West EU: AU (Denmark, Atlantic Continental region), ILVO (Belgium, Atlantic region)
- North-East EU: Luke (Finland, Boreal region), LatHort (Latvia, Nemoral region)

**Figure 1:**
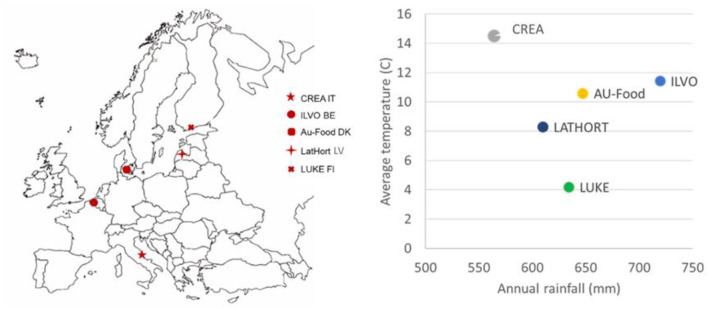
SureVeg experimental sites and related annual rainfalls (mm) and average annual temperatures (°C, 2019).

All experimental fields were under organic vegetable rotation for more than 5 years. In 2018–2019, different vegetable species rotated in a field in each site: reported data are referred to in the second year of the experiment (2019, after 2 years of multi-cropping introduction) (Figure 1).

In each experimental site, the two crops were cultivated under two cropping systems (CS): MC and multi-cropping (namely, IC or SC):

- CREA: faba bean–tomato (SC, three blocks, not fully randomized).
- AU: beetroot–white cabbage (IC, four blocks, fully randomized)
- ILVO: celeriac–leek IC, (four blocks, randomized)
- LatHort: faba bean–white cabbage (SC, three blocks, not fully randomized).
- Luke: onion–white cabbage (IC, four blocks, fully randomized)

In each experimental site, the CS was tested considering three/four blocks, based on field extension and tested CS (IC or SC). A visual representation of the experimental designs applied in each site is reported in Supplementary material 1.

Irrigation was supplied according to plant needs, and fertilization was applied *via* plant-based compost to all the crops. Only at CREA site, MC and SC faba bean was not fertilized to study the local practice of tomato transplanting on flattened legume residues. Details about the pedoclimatic characteristics of EU sites, crop diversification (SC or IC), companion crops, fertilization, irrigation, weed management, sampling time, and other relevant information are reported in Table 1.

**Table 1:** Experimental sites description and management practices implemented.

	**CREA**	**AU**	**ILVO/Inagro**	**LUKE**	**LatHort**
Experimental site	Monsampolo VEgetables organic Long-Term Experiment (MOVE LTE), Monsampolo del tronto (AP, IT)	Aarslev Research Center. Organic since 2013 (Aarslev, DK)	Inagro - Organic experimental farm (Gent, BE)	Luke Mikkeli long-term organic field (Mikkeli, FI)	Pure Research Center of LatHort (Pure, Latvia)
GPS	42° 53′ N, 13° 48′E	55° 18′ N, 10° 27′ E	50°54,312' N, 3°7,646' E	61°41'18.85”N, 27°16'20.17”E	57°02′ N 22°54′ E
Climate	Thermo-Mediterranean (UNESCO/FAO, 1963)	Temperate	Temperate maritime	Continental subarctic (Köppen climate classification)	Temperate - Eastern-continental climate
Total annual precipitation (mm)	564	647	719.6	634	609.8
Average temperatures	Annual 14.5°C	Annual 10.6	Annual 11.43	Annual 4.2	Annual 14.3°C
Soil classification (United States Department of Agriculture, 1996)	Fine-loamy, mixed thermic (Typic Calcixerepts)	Sandy loam (Typic Agrudalf)	Eutric Retisol (Loamic)	Dystric Cambisol	Leached sod-calcareoulus soil
Experimental design	Strip-plot	Randomized split plot	Randomized split plot	Randomized split plot	Nonrandomized split plot
Replications	3	4	4	4	3
Factors	Crop:	Crop:	Crop:	Crop:	Crop:
	i) Faba bean (*Vicia faba* L.), FB	i) Beetroot (*Beta vulgaris* L.), B	i) Celeriac (*Apium graveolens*, L.), CL	i) White cabbage (*Brassica oleraceae* var. capitata f. alba), C	i) Faba bean (*Vicia faba* L.), FB
	ii) Tomato (*Solanum Lycopersicum* L.), T	ii) White cabbage (*Brassica oleraceae*, var. capitata f. alba), C	ii) Leek (*Allium ampeloprasum*, L.), L	ii) Onion (*Allium cepa* L.), O	ii) White cabbage (*Brassica oleraceae* var. capitata f. alba), C
	Cropping system:	Cropping system:	Cropping system:	Cropping system:	Cropping system:
	i) Monoculture (FB-MC; T-MC)	i) Monoculture (B-MC; C-MC)	i) Monoculture (CL-MC; L-MC)	i) Monoculture (C-MC; O-MC)	i) Monoculture (FB-MC; C-MC)
	ii) Bed-by-bed strip cropping (FB-SC; T-SC)**Both crops sampled, being FB-MC and FB-SC not fertilized	iii) Row-by-row Intercropping (IC)	iii) Row-by-row Intercropping (IC)	iii) Row-by-row Intercropping (IC)	iii) Bed-by bed strip cropping (SC)
Row distance (m)	0.7	0.5	0.7	0.5 (Cabbage), 0.5 (onion)	0.7
Plant distance in row (cm)	20 (faba bean), 50 (tomato)	40 (beetroot), 35 (cabbage)	10 (leek), 40 (celeriac)	50 (cabbage), 7 (onion)	50 cabbage, 14 - bean
Plot size (m^2^)	2 × 3.7 (faba bean), 2.8 × 3.7 (tomato)	10 × 4.8	6 × 8	3 × 5	3.5 × 8
Soil tillage practice	Plowing (20-25 cm) and harrowing (faba bean), no tillage (tomato). Faba bean for SC plots was flattened using in line roller crimper	Plowing (20-23 cm), cultivator 8-12 cm depth	Non inversion tillage	Harrow and rototilling (before planting and sowing)	Plowing (22-25 cm depth)
Transplanting	May 13 (tomato transplanting); January 8 (faba sowing)	June 25 (cabbage) June 6 (beetroot sowing)	May 14 (leek) May 15 (celeriac)	May 16 (onion), May 27 (cabbage)	May 31 (cabbage and faba bean)
Crop irrigation	Drip irrigation: 300 L/m^2^ in 25 events	Sprinkler irrigation, 125 mm in 6 events	Spray irrigation: 90 L/m^2^ in three events	Sprinkler irrigation: 15 mm on event	Manual irrigation: 47 L/m^2^ in five events
Fertilization	Faba bean MC and SC: not fertilized. Tomato MC and SC: on May 11, 2019, flattened faba bean residues; on May 16, 2019, Compost at 11.5 Mg ha^−1^	Cabbage and beetroot MC and IC fertilized: May 29: fresh clover; Cabbage MC: 24 Mg ha^−1^, beetroot MC: 26 Mg ha^−1^; SC: 24 Mg ha^−1^, October 3, 2019 Compost: 10 Mg ha^1^ in all cropping system	Celeriac and leek MC and IC fertilized: April 2: Haspargit Potassium fertilizer 667 kg ha^−1^ April 20, 2019: organic granular fertilization (11-0-5), 500 kg/ha OPF April 25, 2019: green compost, 12 ton/ha	Cabbage and onion MC and IC fertilized: 1 October Wood based soil improver; Cabbage MC; 59 Mg ha^−1^; onion MC; 11 Mg ha^−1^; SC: 30 Mg ha^−1^, May and July 2019: Biokali cabbage MC, 10 Mg ha^−1^; onion MC: 1.9 Mg ha^−1^; SC: 9 Mg ha^−1^	Faba bean and cabbage MC and SC fertilized: May 3 2019: before trial establishment with green compost at 50 t ha^−1^
Weeding	Weed cutting with mowing blade before sowing; manual weeding during cropping cycle	Interrow weeding with weed brush machine (Rath Maschinen, Germany) - late June to early Aug (5 times) Manual weeding	Mechanical weeding from late May to late Sept. Precision arrow (5 times), ridging (3 times on leek), hoeing (5 times on celeriac)	Week 23 and 24, 17.-18.6., 2.-5.7. (three times), hand weeding and harrowing	Late June to early Aug, manual weeding during cropping cycle (3 times)
Sampling time	Bulk soil: Late July (faba bean dry grains harvest); at half August (tomato harvest)	Bulk soil: Late October (after cabbage and beetroot harvest)	Bulk soil: Late October	Bulk soil: 2018 in late September	Bulk soil: Early June
	Rhizosphere soil: at late July (faba bean dry grains harvest); at half August (tomato harvest)	Rhizosphere soil (only in MC systems): late August 29 (close to harvest)	Rhizosphere soil: mid-October	None	None

### Soil sampling and physicochemical parameters

In 2019, at crop harvest, the bulk soil was sampled at 0–30 cm layer in all field trials. In AU, ILVO, and Luke sites, IC soil sampling was carried out in-between the changing rows, while at CREA and LatHort sites, SC soil sampling was performed in-between the beds' external rows. Only at CREA site, SC soil samples were collected in FB and T SC beds' external rows, being nonfertilized the soil under faba bean and fertilized that under tomato. In MC and IC/SC, after collecting four subsamples in each block, one composite soil sample/block was analyzed. The pH, N_tot_, P_av_, HWC, HWP, C_mic_/TOC, C_mic_, N_mic_, and P_mic_ were determined at CREA, AU, and ILVO sites, while soil HWC and HWP were assessed at all the sites.

Soil moisture content was determined as the weight loss at 105°C. The pH was determined in 1M KCl (1/5 v/v), while the bulk density (in g cm^−3^) by collecting undisturbed soil cores (100 cm^3^) using an auger at depth of 0–30 cm layer (ISO 11272). Total organic C (TOC, in %) was obtained by dry combustion method at 1,050°C using LECO TOC Analyzer (mod. RC-612; LECO Corporation, 1987), after subtracting inorganic C (in %), while total N (in g kg^−1^) by dry combustion method. To calculate P_av_ (in mg kg^−1^), dried soil samples at a ratio 1:10 (v/v) in CaCl_2_ solution (NEN 5704, at ILVO site) or Melich 3/Olsen solution (Mehlich, 1984; Ziadi et al., 1993, at AU and CREA sites) were extracted for 2 h and then the extracts were analyzed by simultaneous plasma emission spectrophotometer (ICP-OES Iris; Thermo Optek) or by Skalar SAN++ CFA. In LatHort plant available, phosphorus was detected by using calcium lactate according to the Egner–Riehm method (Egner et al., 1960). Soil C (HWC) and soil P (HWP) soluble in hot water (in mg kg^−1^) were extracted using Haynes and Francis method (Haynes and Francis, 1993), by extraction of 5 g of dried soil samples in 25 ml of demineralized water for 16 h in a hot water bath at 70°C. After centrifugation and filtration on Machery–Nagel mn640d filter, the total C was determined by dry combustion by LECO TOC Analyzer or Skalar Primacs SLC TOC-analyzer. HWP was measured on water extracts by ICP-OES (ICP-OES Iris or Thermo Optek VISTA-PRO, Varian, Palo Alto, CA) or by Skalar SAN++ CFA.

### Soil microbial biomass stoichiometry

Soil microbial biomass stoichiometry was assessed at CREA, AU, and ILVO sites by determination of microbial biomass C (C_mic_), N (N_mic_), and P (P_mic_) content in the bulk soil collected in MC and IC plots (Zhang and Elser, 2017), respectively. On saturated soil samples sieved at 2 mm, soil water holding capacity (WHC) at −33 kPa (pF = 2.5) was determined using a pressure cell apparatus. Preincubation until reaching 60% of WHC for 10 days at 30°C was performed (Vance et al., 1987). Incubated soil samples were then divided into two subsamples, and one of them was fumigated with ethanol-free chloroform (CHCl_3_) under vacuum overnight. To measure C_mic_ and N_mic_, both fumigated and unfumigated soils were extracted with 0.5 M K_2_SO_4_ solution (soil/extraction solution: 1:4) at room temperature for 30 min (Brookes et al., 1985; Voroney et al., 2008). C_mic_ and N_mic_ were measured on filtered extracts using Shimadzu TOC-V-CSN analyzer (Fornasier et al., 2014). P_mic_ was determined after extraction of fumigated and not fumigated soils with 0.5 M NaHCO_3_ solution, using ammonium molybdate–stannous chloride colorimetric method (Sparling et al., 1985), and then analyzed by a continuous-flow colorimeter Autoanalyser Technicon II or Flowsys Analyzer SYSTEA S.p.A. (absorbance of the extracted solution at λ = 882 nm). Microbial coefficient (C_mic_/TOC) was calculated and used as ecological indicator of environmental changes induced by different CS or soil managements (Anderson, 2003; Moscatelli et al., 2005). C_mic_/N_mic_ and N_mic_/P_mic_ ratios were then determined to evaluate the potential shift of the soil microbiome toward bacteria or fungi community (Strickland and Rousk, 2010; Mouginot et al., 2014).

### Crop root mycorrhizatin

At CREA, AU, and LatHort sites, crop roots were sampled in the field, considering four plant per treatment per block. Roots were separated from the soil by washing the sampled material under fresh water in a sieve of 0.5 mm mesh, then divided into first, second, and third orders' lateral roots. Collected third-order lateral roots (diameter < 2 mm) were stained by immerging them in a stain solution of 0.05% w/v methyl blue in lactoglycerol (1:1:1 lactic acid/glycerol/distilled water) for 1 min, and then distained in bi-distilled water for 1 min more (Grace and Stribley, 1991). A total of 10 × 1 cm root segment (third-order lateral fine roots) per plant were selected at random from the stained root segments, by cutting them with a razor blade from 5 to 15 mm from the root tip. The total number of observed root segments per each treatment was: 10 × 4 plants × 3/4 blocks = 120/160 segments. The evaluation of mycorrhizal colonization intensity (M%) of each segment was assessed under a light microscope (Nikon E100 at 10 × and 40 ×). The mycorrhizal infection score (Trouvelot et al., 1986) was calculated by attributing to each root fragment increasing scores from 0 to 5, applying the following formula: M_i_ % = (95n_5_ + 70n_4_ + 30n_3_ + 5n_2_ + n_1_)/total number of observed segments, where n5 is the number of segments rated 5, n_4_ is the number of segments rated 4, and so on.

### Rhizosphere bacterial and fungal community

To evaluate bacteria and fungi diversity as affected by multi-cropping at CREA and ILVO sites, four rhizosphere soil samples per each MC and IC/SC plot were collected. In AU system, bacteria and fungi diversity were evaluated by NGS only in MC, collecting five rhizosphere soil samples per each plot. Rhizosphere soil sampling was performed using the method proposed by Lundberg et al. (2013), considering rhizosphere soil as extending up to 1 mm from the root surface. Collected roots from the soil sampled using a stainless cylinder were placed in a sterile 50 ml tube containing 25 ml phosphate buffer, vortexed at maximum speed for 15 s, to release most of the rhizosphere soil from the roots. The turbid suspension was then filtered through a 100 nm nylon mesh cell strainer into a new 50 ml tube to remove plant parts and large sediment particles, and then further centrifuged for 15 min at 3,200 rpm to form a loose pellet containing fine sediment and microorganisms. Phosphate buffer was removed, and the resulting rhizosphere pellets (250 mg) were used for DNA extraction with the PowerSoil DNA isolation kit (Qiagen) according to the manufacturer's instructions. DNA was stored at −20°C. The extracted DNA was used for identifying bacterial (V3–V4 16S rRNA gene) and fungal rhizosphere populations (ITS2) through amplicon sequencing using Illumina technology (Illumina, San Diego, CA, USA) by Admera, United States.

Libraries were constructed following Illumina 16S Metagenomic Sequencing Library Preparation protocol in two amplification steps: an initial PCR amplification using locus-specific PCR primers and a subsequent amplification that integrates relevant flow cell-binding domains and unique indices (NexteraXT Index Kit, FC-131-1001/FC-131-1002; Illumina Inc., San Diego, CA, USA).

For the first PCR step, 16S rRNA gene S-D-Bact-0341-b-S-17 (5′-CCTACGGGNGGCWGCAG-3′) and S-D-Bact-0785-a-A-21 (5′-GACTACHVGGGTATCTAATCC-3′) primers were used (Klindworth et al., 2013; Debode et al., 2016) and for ITS fungal rDNA-ITS2 region fITS7b (5′-GTGAATCATCRAATYTTTG-3′) from Ihrmark et al. (2012) and the ITS4NGSr (5′-TTCCTSCGCTTATTGATATGC-3′) primer (Tedersoo et al., 2014) (Supplementary material 2). The amplicons were sequenced on Illumina MiSeq 2 × 300 bp paired-end platform (Illumina Inc., San Diego, CA, USA).

### Bioinformatic elaboration and statistical analysis

Soil physicochemical (pH, bulk density, TOC, N_tot_, P_av_, HWC, and HWP) and biochemical parameters (C_mic_, N_mic_, P_mic_, C_mic_/TOC, C_mic_/N_mic_, and N_mic_/P_mic_) and root mycorrhizal colonization intensity (M) were analyzed by one-way ANOVA, as affected by CS, after verification by Shapiro–Wilk normality test. In CREA system, since both the crops were sampled separately under SC management, CS and crop effect were considered, together with their interaction. Shapiro–Wilk test was performed to check the normality of the data. *Post hoc* Tukey's HSD test was carried out to compare means using SPSS (IBM Corp., Armonk, NY, United States).

The principal component analysis (PCA) on soil physicochemical and biochemical indicators at AU, CREA, and ILVO experimental sites was produced using the R package factoextra.

Raw paired-end reads were processed using the R package DADA2 version 1.16.0. The Divisive Amplicon Denoising Algorithm (DADA) is based on the identification of single nucleotide sequence variants producing an amplicon sequence variant (ASV) table with a higher resolution of the traditional OTU table. The confidence level for an assignment was set at 3%, as a standard procedure with the DADA2 pipeline (97% identity level to define taxonomic units).

The Silva v132 database and the UNITE v020219 database were used to assign taxa for bacteria and fungi, respectively.

At AU, CREA, and ILVO experimental sites, the α-diversity of bacterial and fungal communities was determined by calculating richness, evenness, Shannon index, and Simpson indexes. Species richness is a measure for the total number of species in the community. Evenness refers to how close in numbers each species is in an environment.

The results of the soil microbiome were shown only with at least 0.1% relative abundance in the whole dataset (Wassermann et al., 2019). In AU site, only the crop effect was studied.

Within each condition, the normal distribution of data was verified using the Shapiro–Wilk test in R (V3.4.4), then applying ANOVA test and Tukey's *post hoc* test to compare data as affected by considered factors.

We also explored differences in the relative abundance of some fungal families that also include pathogenic fungi, particularly *Scleroniaceae, Olpidiaceae*, and *Nectriaceae*. We used permutational multivariate analysis of variance (PERMANOVA) on the experimental sites. Nonmetric multidimensional scaling (NMDS) was performed on CREA and ILVO datasets with the Bray–Curtis dissimilarity index, using two dimensions (*k* = 2). These analyses were carried out using the *vegan* package.

Finally, we tested the effect of crop, block, and CS on the relative abundance of *Fusarium* spp. at ILVO and CREA, while we tested only the effects of crop and block on *Olpidium brassicae* at AU. Beta-regression models were used (*betareg* package). Shapiro–Wilk test was used to test the normality of residuals. Differences between treatment means were compared using a *post-hoc* Tukey test (α = 0.05) (*emmeans* package). Those statistical analyses were performed in R (version 4.0.3, 2020).

## Results

### Soil physicochemical parameters and microbial biomass stoichiometry

**Figure 2:**
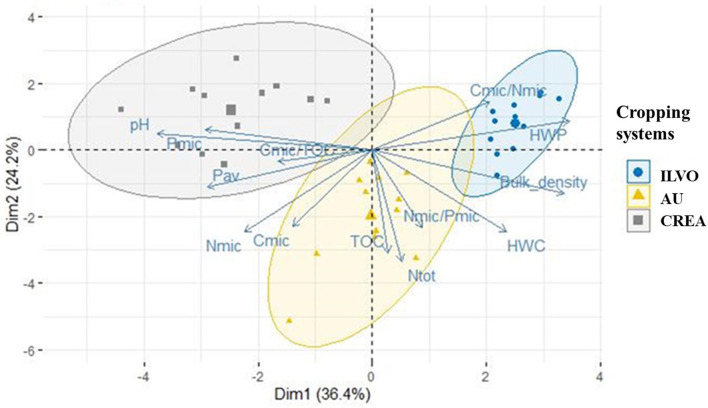
Principal component analysis (PCA) biplot and confidence ellipses ordering the Belgian, Danish, and Italian experimental sites in relation to relevant soil physicochemical and biochemical parameters (bulk density, pH, N_tot_, P_av_, HWC, HWP, C_mic_/TOC, C_mic_, N_mic_, P_mic_, C_mic_/N_mic_, and N_mic_/P_mic_) at harvest.

The principal component analysis, applied to soil physicochemical (bulk density, pH, N_tot_, P_av_, HWC, HWP) and biochemical indicators (C_mic_/TOC, C_mic_, N_mic_, P_mic_, C_mic_/N_mic_, N_mic_/P_mic_) in AU, CREA, and ILVO, did not discriminate across CS. Conversely, Figure 2 shows that “site” was a discriminant factor regardless of the CS applied.

Indeed, principal components 1 and 2 explained 60.6% of variability and strongly discriminated across the CREA, ILVO, and AU systems. Along with the positive value of component 1, ILVO site was associated with bulk density, HWP, and C_mic_/N_mic_ indicators, while CREA site to pH, P_av_ and P_mic_ content, and C_mic_/TOC. The component 2 (Y-axis) affected mainly the AU beetroot site in terms of TOC and total N (N_tot_), while both the components explained the variability of C_mic_, N_mic_, N_mic_/P_mic_ ratio, and HWC. PCA analysis evidenced that the three sites, characterized by different soil parameters, must be evaluated separately to highlight the effect of multi-cropping management on considered microbial indicators.

In Table 2, the same soil physicochemical and microbial parameters recorded at crop harvesting are reported, as affected by CS. At CREA only, the crop effect was also evaluated, with related interaction.

**Table 2:** Soil chemical, physical and stoichiometric parameters at CREA, AU, and ILVO sites in 2019, namely: pH, bulk density (g cm^−3^), total organic C (TOC, %), total N (g kg^−1^), available P (mg kg^−1^), C_mic_, N_mic_, P_mic_ content, plant mycorrhizal colonization intensity (M%), microbial coefficient (C_mic_/TOC), C_mic_/N_mic_ and N_mic_/P_**mic**_ ratios at crop harvesting.

	**pH**	**Bulk density**	**TOC**	**N_tot_**	**Pav**	**Cmic**	**Nmic**	**Pmic**	**Cmic/TOC**	**Cmic/Nmic**	**Nmic/Pmic**
***Units***		***g cm^−3^***	***%***	***g kg^−1^***	***mg kg^−1^***	***mg kg^−1^***	***mg kg^−1^***	***mg kg^−1^***			
**Faba bean–tomato (CREA, IT)**
**FB-MC**	7.80 ± 0.05b	1.24 ± 0.01b	1.12 ± 0.02	1.03 ± 0.02a	34.0 ± 6.1a	51 ± 32b	10.6 ± 7.5 c	3.2 ± 0.6b	0.46 ± 0.29b	12.9 ± 11.6	3.8 ± 0.6b
**T-MC**	7.82 ± 0.04b	1.35 ± 0.03a	1.09 ± 0.07	0.73 ± 0.04b	16.3 ± 6.4b	220 ± 61a	37.5 ± 3.2a	3.3 ± 0.5b	2.03 ± 0.25a	5.9 ± 6.3	11.6 ± 1.5a
**FB-SC**	7.90 ± 0.03a	1.30 ± 0.04a	1.05 ± 0.05	1.20 ± 0.03a	44.4 ± 1.6a	87 ± 24b	6.2 ± 2.9c	4.8 ± 1.1 ab	0.82 ± 0.59b	12.5 ± 5.9	9.6 ± 1.4ab
**T-SC**	7.79 ± 0.04b	1.15 ± 0.06b	1.08 ± 0.10	0.73 ± 0.02 ab	22.4 ± 4.0ab	195 ± 57a	28.5 ± 9.5b	4.5 ± 0.4a	1.79 ± 0.37a	7.0 ± 0.4	6.6 ± 2.7b
C-effect	n.s.	n.s.	n.s.	n.s.	^*^	^***^	^***^	n.s.	^***^	n.s.	n.s.
CS-effect	^*^	n.s.	n.s.	n.s.	n.s.	n.s.	n.s.	^*^	n.s.	n.s.	^*^
C × CS	n.s.	^*^	n.s.	^**^	^**^	n.s.	^**^	^***^	n.s.	n.s.	^**^
**Cabbage–beetroot (AU, DK)**
**C-MC**	6.70 ± 0.10	1.47 ± 0.03	1.75 ± 0.04	1.67 ± 0.07	29.7 ± 0.4	121 ± 32b	16.8 ± 4.4	4.0 ± 1.8	0.69 ± 0.14b	7.8 ± 3.5	5.51 ± 3.8
**B-MC**	6.70 ± 0.09	1.46 ± 0.01	1.75 ± 0.02	1.58 ± 0.04	31 ± 0.2	181.9 ± 45a	24.1 ± 11.4	3.5 ± 1.4	1.05 ± 0.27a	8.1 ± 1.9	7.16 ± 1.9
**IC**	6.60 ± 0.11	1.47 ± 0.02	1.75 ± 0.06	1.58 ± 0.05	32.5 ± 0.2	177.6 ± 98a	27.6 ± 27	3.0 ± 2.0	1.04 ± 0.59a	8.55 ± 3.5	15.77 ± 14.0
CS-effect	n.s.	n.s.	n.s.	n.s.	n.s.	^*^	n.s.	n.s.	^*^	n.s.	n.s.
**Leek–celeriac (ILVO, BE)**
**L-MC**	5.60 ± 0.04	1.55 ± 0.04	1.16 ± 0.02	1.07 ± 0.05	3.6 ± 0.3	120 ± 44	11.71 ± 6.4	0.68 ± 0.2b	1.02 ± 0.2	10.2 ± 0.7.2	18.70 ± 1.10 a
**CL-MC**	5.70 ± 0.01	1.54 ± 0.02	1.14 ± 0.05	1.04 ± 0.04	4.0 ± 0.5	105 ± 27	8.32 ± 9.5	1.33 ± 0.9 ab	0.9 ± 0.4	30.5 ± 12.9	5.25 ± 0.24b
**IC**	5.70 ± 0.06	1.53 ± 0.02	1.15 ± 0.05	1.07 ± 0.03	3.9 ± 0.5	119 ± 44	4.8 ± 2.5	2.33 ± 0.6a	1.03 ± 0.4	27.5 ± 11.3	2.40 ± 0.21c
CS-effect	n.s.	n.s.	n.s.	n.s.	n.s.	n.s.	n.s.	^*^	n.s.	n.s.	^*^

Levels of statistical significance (p value) are: ^*^p < 0.05, ^**^p < 0.01, and ^***^p < 0.001, n.s., not significant (ANOVA).

Different letters represent significant differences (Tukey's HSD test for means comparison).

At CREA, we found a CS effect on pH, which was significantly higher under faba bean SC (7.90) compared to faba bean MC (7.80, *p* = 0.05023). Soil bulk density and N_tot_ were affected by the crop × CS interaction. Similarly, P_av_ varied across crops and CS, being the lowest one in tomato MC (16.3 mg kg^−1^) and the highest in faba bean MC and SC (34.0 and 44.4 mg kg^−1^, respectively), due to the crop × CS interaction. C_mic_ was affected only by crop type, while N_mic_ was affected by the interaction between crop type and CS. We also found a significant CS effect and crop × CS interaction on P_mic_. SC showed a four-fold increase under faba bean SC (P_mic_ = 12.2 mg kg^−1^) compared to MC (P_mic_ = 3.3 mg kg^−1^). C_mic_/TOC was instead significantly affected by the crop type. Anyway, an interaction effect of crop × CS was found on soil bulk density, N_tot_ and P_av_, N_mic_, P_mic_ and consequently, on N_mic_/P_mic_. This last ratio was lower in both the faba bean and tomato SC, and higher under tomato. N_mic_/P_mic_ was interestingly changed by CS, increasing in faba bean (from 3.8 to 9.6 mg kg^−1^) and decreasing in tomato, going from MC to SC (from 11.6 to 6.6. mg kg^−1^).

In the AU site, CS affected significantly C_mic_ and C_mic_/TOC only, with the lowest found under MC cabbage (120.9 and 6.92 mg kg^−1^, respectively).

At ILVO, a significant effect of CS was found on P_mic_ and N_mic_/P_mic_. Here, the lowest P_mic_ content and the highest N_mic_/P_mic_ content were found under MC leek (0.68 and 18.7 mg/kg, respectively).

**Figure 3:**
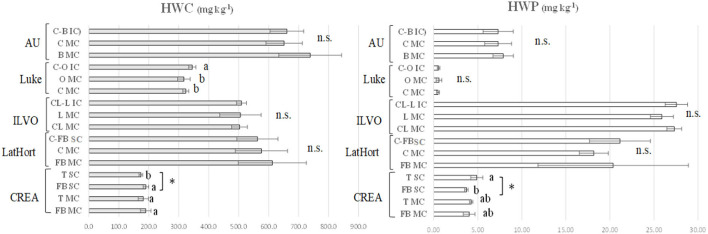
Soil HWC and HWP (mg kg^−1^) in: monocropped faba bean (FB MC) and tomato (T MC), strip cropped faba bean (FB SC) and tomato (T SC) at CREA; monocropped faba bean (FB MC) and cabbage (C MC), strip cropped faba bean and cabbage (FB-C IC) at LatHort; monocropped celeriac (CL MC) and leek (L MC), intercropped celeriac and leek (CL-L IC) at ILVO; monocropped cabbage (C MC) and onion (O MC), intercropped cabbage and onion (C-O IC) at Luke; monocropped beetroot (B MC) and cabbage (C MC), and intercropped beetroot and cabbage (B-C IC) at AU. Levels of statistical significance (*p* value) are: *p < 0.05, **p < 0.01, and ***p < 0.001, *ns* = not significant (ANOVA). Different letters represent significant differences (Tukey's HSD test for means comparison).

In Figure 3, HWC and HWP, measured in bulk soil of CREA, AU, ILVO, Luke, and LatHort experimental sites by comparing the MC vs. IC/SC systems, are reported.

In CREA system, the lowest HWC was recorded in tomato SC (173.5 mg kg^−1^), while in faba bean and tomato MC (189.7 and 181.5 mg kg^−1^), and in faba bean SC (190.0 mg kg^−1^), they were the highest ones. In AU experiment, no differences in soil HWC were observed in beetroot and cabbage MC compared to IC system. On the opposite, at Luke, cabbage and onion MC gave significantly lower HWC values (323.5 and 317.0 mg kg^−1^) compared to cabbage–onion IC (345.1 mg kg^−1^). Both at ILVO and LatHort system, no significant difference was observed between MC and IC/SC.

HWP was strongly affected by the experimental sites, being highest values recorded in ILVO and LatHort systems compared to AU, Luke, and CREA systems. In considered experimental trials, no significant effect of IC on HWP was found, except at CREA faba bean–tomato system, where soil HWP was significantly higher under tomato SC (4.90 mg kg^−1^) compared to faba bean SC (3.72 mg kg^−1^); intermediate values were recorded under faba bean and tomato MC (4.03 and 4.26 mg kg^−1^, respectively).

A correlation (*R* = 0.6188) was found between C_mic_/TOC and HWC, as a result of different pedoclimatic location of considered case studies.

### Crop root mycorrhization

**Figure 4:**
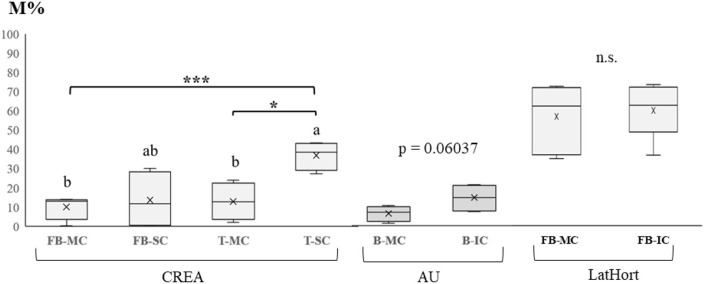
Mycorrhizal colonization intensity (M%) recorded in **(A)** monocropped and strip cropped faba bean (FB-MC, FB-SC) and monocropped and strip cropped tomato (T-MC) and strip cropped, T-SC) (CREA, IT); **(B)** beetroot monocropping (B-MC) and intercropping (B-IC) systems (AU, DK); **(C)** faba bean in monocropping (FB-MC) and intercropping (FB-IC) systems (LatHort, LV). Levels of statistical significance are: *p < 0.05, **p < 0.01, and ***p < 0.001, *ns*, not significant (ANOVA). Different letters represent significant differences (Tukey's HSD test for means comparison).

In Figure 4, the mycorrhizal colonization intensity (M%) recorded at CREA, AU, and LatHort systems is reported, as affected by SC and IC, compared to MC.

At CREA, a significant increase in crop root mycorrhization was observed in tomato SC, with an average value three times higher than that recorded in tomato MC, going from 12.5% to 37.2%. No CS effect was observed between faba bean MC and SC. In AU, even if both cabbage and beetroot are recognized as nonmycorrhizal plant species, we found a highest M% in IC beetroot (13.8%), although not significantly different from MC beetroot (6.3%; *p* =0.06037).

### Rhizosphere bacterial and fungal community

**Figure 5:**
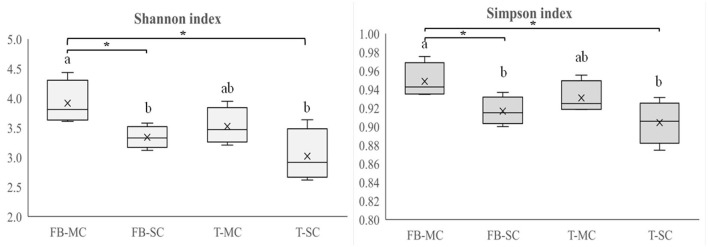
Fungal community diversity indices (Shannon index, Simpson index) recorded in faba bean (FB) and tomato (T) monocropping (MC) and strip cropping (SC) at CREA site. Levels of statistical significance (*p* value) are: ^*^p < 0.05, ^**^p < 0.01, and ^***^p < 0.001, *ns*, not significant (ANOVA). Different letters represent significant differences (Tukey's HSD test for means comparison).

Rhizosphere bacterial and fungal community diversity of each crop was studied by calculating richness, evenness, Shannon index, and Simpson index at CREA and ILVO sites, as affected by either IC or SC, while at AU only on MC. In CREA system, significant differences were observed only in fungal community composition (Figure 5).

The crop × CS interaction was significant at CREA. Shannon index gave the lowest value in tomato SC and the highest under faba bean MC. Similarly, Simpson index was the lowest under tomato SC and the highest under faba MC. Fungi richness was the highest under faba bean MC, and the lowest under faba bean SC.

**Figure 6:**
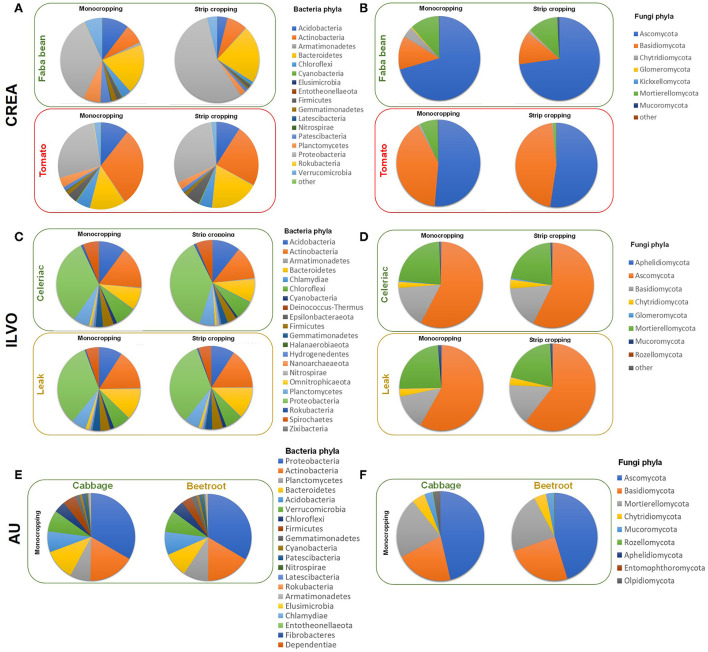
The relative abundance of bacteria and fungi phyla recorded in CREA [**(A)** = bacteria phyla; **(B)** = fungi phyla], ILVO [**(C)** = bacteria phyla; **(D)** = fungi phyla], and AU [**(E)** = bacteria phyla; **(F)** = fungi phyla] experimental sites were calculated, considering the crop type and the cropping systems (in AU site, only the crop effect is reported).

In Figure 6, the relative abundance of bacteria and fungi phyla as affected by crop and CS at CREA, ILVO sites and at AU (here, on MC only) are reported (see also Supplementary material 3).

At CREA site, bacteria community obtained after bacterial rDNA 16S sequencing of rhizosphere soil samples revealed 10,025 different ASV. The taxonomic assignment revealed 9 unidentified ASV, while the others were assigned to 30 different bacteria Phyla (SM1). After the taxonomic assignment at CREA site 264 family and 585 genera were identified. The most represented ASV were *Proteobacteria* (31.0%), followed by *Bacteroitedetes* (13.2%), *Actinobacteria* (10.0%), *Acidobacteria*, (10.0%), *Planctomycetes* (8.9%), *Chloroflexi* (7.3%), *Verrucomicrobia* (6.2%), *Patescibacteria* (3.7%), *Firmicutes* (3.2%), *Gemmatimonadetes* (2.6%), *Cyanobacteria* (1.1%), *Armatimonadetes* (1.0%), and unindentified (1.6%) (Figure 6A).

The highest relative abundance associated with faba bean MC and SC was observed for *Proteobacteria* (35.4% and 55.5% in MC and SC, respectively; *p* = 0.022), *Bacteroidetes* (19.1and 21.8%), *Acidobacteria* (10.5% and 4.0%, *p* = 0.0524), and *Actinobacteria* (7.6% and 7.8%), being a relative abundance of other bacteria Phyla lower than 7.0% (Figure 6A). Under tomato, again *Proteobacteria* were the most abundant phyla (26.8and 29.6% in MC and SC, respectively), followed by *Actinobacteria* (29.5% in MC and 23.5% in SC, respectively; *p* = 0.0491) and *Bacteroitedes* (13.5% in MC and 18.7% in SC) (Figure 6A).

The overall fungal community obtained after rDNA ITS fungal sequencing of IT rhizosphere soil samples revealed 1,606 different ASV. The taxonomic assignment revealed 20% of unidentified ASV, while the others were assigned to 14 different Phyla (Supplementary material 3), particularly *Ascomycota* (44%), *Basidiomycota* (15%), *Chytridiomycota* (8%), *Glomeromycota* (4%), *Mortierellomycota* (3.2%), *Mucoromycota* (1%), and *Kickxellomycota* (0.4%), 156 Family and 245 Genera.

Under faba bean, *Ascomycota* were overrepresented in both MC and SC (70.7% and 72.8%, respectively), followed by *Basidiomycota* (12.9% and 12.9%), *Mortierellomycota* (11.5% and 12.1%), and *Chytridiomycota* (4.2%, and 1.0%) (Figure 6B). Under tomato, *Ascomycota* were the most represented Phyla (51.4% and 52.5%, in MC and SC, respectively), followed by *Basidiomycota* (40.6% and 45.7%). A reduction in relative abundance of *Mortierellomycota* was recorded under SC (7.0% in MC and 1.4% in SC, respectively; *p* = 0.0646). Most of the ASV identified in *Mortierellomycota* Phylum at CREA site belongs to *Mortierella* genus.

Regarding ILVO site, the bacterial rDNA 16S sequencing of rhizosphere soil samples revealed 15,334 different ASV describing the overall bacteria community. The taxonomic assignment revealed 2.5% of unidentified ASV, while the others were assigned to 31 different bacteria Phyla, 264 Family, and 585 Genera (Supplementary material 3). The celeriac–leek system was dominated by *Proteobacteria* (28.6%), followed by *Planctomycetes* (14.0%), *Bacteroitedes* (11.9%), *Acidobacteria* (9.7%), *Verrucomicrobia* (7.9) *Actinobacteria* (7.6%), *Chloroflexi* (4.8%), *Firmicutes* (4.6%), *Patescibacteria* (3.15%), *Gemmatimonadetes* (2.55%), *Chlamydiae* (1.47%), *Cyanobacteria* (1.31%), and other Phyla being lower than 1.0%.

Under celeriac, *Proteobacteria* showed the highest relative abundance in both MC and SC (33.1% and 37.6%, respectively), followed by *Actinobacteria* (16.0% in MC and 12.6% in IC, respectively; *p* = 0.0488), *Acidobacteria* (10.2% and 10.7%) and *Bacteroidetes* (8.4 and 8.9%) (Figure 6C).

Under leek, again *Proteobacteria* showed the highest relative abundance in MC and IC (33.3 and 33.6%, respectively), followed by *Actinobacteria* (15.5% and 15.6%), *Bacteroidetes* (12.3 and 12.2%) and *Acidobacteria* (9.1% and 9.2%) (Figure 6C). A crop effect emerged on *Bacteroidetes* relative abundance, highest under leek when compared with celeriac (12.2% and 8.6%, as MC vs. IC averages, respectively), independently from the CS (*p* = 0.0029).

The rDNA ITS fungal sequencing of ILVO rhizosphere soil samples revealed 3,496 different AVS describing the overall fungi community. The taxonomic assignment revealed 30% of unidentified ASV, while others were assigned to 14 different fungi Phyla, 190 Family and 332 Genera (Supplementary material 3), where *Ascomycota* dominated (36.8%), followed by *Basidiomycota* (15.5), *Chytridiomycota* (7.6%), *Glomeromycota* (3.6%), *Mortierellomycota* (2.0%.), *Aphelidiomycota* (1.3%), *Mucoromycota* (1.2%), and other Phyla with a percentage of ASV lower than 1%.

Under celeriac MC and IC, more than half of fungi population was constituted by *Ascomycota* (57.5 and 57.2%, respectively), followed by *Mortierellomycota* (22.9% and 21.6%) and *Basidiomycota* (16.0 and 16.2%) (Figure 6D). Under leek, *Ascomycota* Phylum was overrepresented (58.0 and 60.5%, in MC and IC, respectively), followed by *Mortierellomycota*, which decreased in IC (23.9 and 20.3%, respectively; *p* = 0.0672) and *Basidiomycota* (13.4 and 15.0%) (Figure 6D).

In AU site, reported NGS results refer only to cabbage and beetroot MC, describing the microbial community composition under the two companion crops used for field trial. Overall bacteria community obtained after bacterial rDNA 16S sequencing of rhizosphere soil samples revealed 38,541 different ASV. The taxonomic assignment revealed 855 (2.2%) unidentified ASV, while the others were assigned to 30 different bacteria phyla (SM1). One of them was the former candidate lineages frankia bacterial peritonitis (FBP) recently accepted as the novel phylum *Abditibacteraeota* (Tahon et al., 2018), and five of them belong to new candidates' phyla. After the taxonomic assignment in AU system, the highest number of ASV identified was of *Proteobacteria* (24.9 %), mostly constituted by gamma-proteobacteria (43.4% of the ASV identified as *Proteobacteria*), followed by delta-proteobacteria (39.3%) and alpha-proteobacteria (16.8%). The other Phyla identified were *Planctomycetes* (11.5%), *Acidobacteria* (11.5%), *Bacteroidetes* (10.1%), *Verrucomicrobia* (9.2%), *Patescibacteria* (7.7%), *Actinobacteria* (6.6%), *Chloroflexi* (4.5%), *Gemmatimonadetes* (2.9%), *Chlamydiae* (1.7%), *Firmicutes* (1.4%), and *Latescibacteria* (1.0%), being unidentified phyla <1% of identified ASV.

All the ASV belongs to 292 different Family and 672 Genera. In AU experimental site, the relative abundance associated with beetroot and cabbage for *Proteobacteria* and *Actinobacteria* were similar (in beetroot: 33.5% and 16.5%; in cabbage: 33.3% and 17.0% respectively, Figure 6E). *Planctomycetes, Acidobacteria, Chloroflexi*, and *Gemmatimonadetes* gave slightly higher relative abundances under beetroot (9.3, 8.6, 4.7, and 1.9%, respectively) than under cabbage (7.6, 7.7, 4.3, and 1.6%, respectively). Relative abundance of *Verrucomicrobia* also was slightly higher under beetroot (8.1%) than under cabbage (7.9%) (Figure 6E).

The phylum *Bacteroidetes, Firmicutes*, and *Patescibacteria* showed a higher relative abundance under cabbage (11.4, 4.8, and 1.3%, respectively) then under beetroot (9.1, 3.8, and 1.0%, respectively, Figure 6E). The relative abundance observed for the other Phyla was lower than 1.0%.

The overall community obtained after rDNA ITS fungal sequencing of rhizosphere soil samples revealed 6,546 different ASV. The taxonomic assignment revealed a very huge number of unidentified ASV (1870). The others were assigned to 14 different fungi Phyla (Supplementary material 3), particularly *Ascomycota* (39.6%), *Basidiomycota* (16.2%), *Chytridiomycota* (7.0%), *Glomeromycota* (3.5%), *Mortierellomycota* (2.0%), *Aphelidiomycota* (1.1) *Mucoromycota* (0.8%) *Rozellomycota* (0.5%), *Entomophthoromycota* (0.3%), *Olpidiomycota* (0.1%), and *Kickxellomycota* (0.2%), 251 Family and 481 Genera. The highest relative abundance was associated *Ascomycota* under cabbage (46.3%), being 45.2% under beetroot (Figure 6F). Opposite results were observed for *Basidiomycota* where the relative abundance was higher in beetroot samples (24.6%) than in cabbage samples (21.0%). The relative abundance of *Mortierellomycota* was 22.3% in beetroot and 21.8% in cabbage samples (Figure 6F). *Chytridiomycota* had a relative abundance of 4.6% and 4.9%, while *Mucoromycot*a 2.9% and 3.0% under beetroot and cabbage, respectively (Figure 6F). Regarding *Olpidiomycota* phylum, it was interesting that <36 counts only in two replicates of the samples were observed in beetroot samples, while over 550 counts for cabbage, corresponding to 0.0% of relative abundance under beetroot and 2.7 % under cabbage. For *Aphelidiomycota* and *Entomophthoromycota*, we observed an opposite trend compared to *Olpidiomycota*, with 0.0% of relative abundance under cabbage and 0.1% under beetroot for both phyla.

To better highlight the similarity–dissimilarity of bacterial and fungal populations in the investigated systems, in **Figure 8**. Venn diagrams of overall bacteria and fungi phyla recorded in CREA, ILVO, and AU experimental sites are reported.

**Figure 7:**
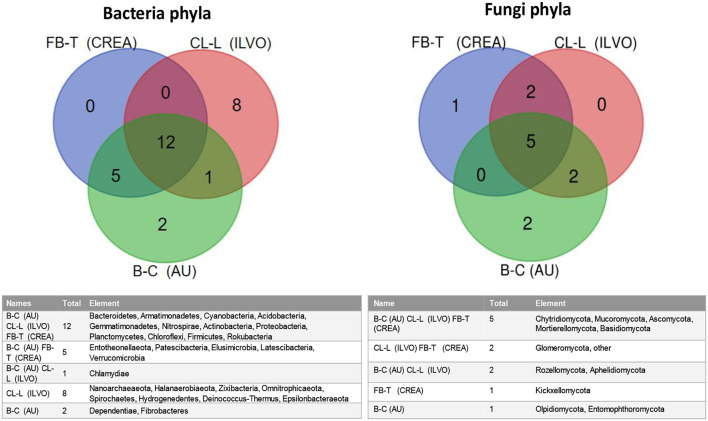
Venn diagrams of bacteria and fungi phyla in CREA, ILVO, and AU experimental field experiments. IN AU system, data are referred only to beetroot and cabbage MC systems.

*Proteobacteria, Actinobacteria, Acidobacteria, Bacteroidetes, Armatimonadetes, Cyanobacteria, Gemmatimonadetes, Nitrospirae, Planctomycetes, Chloroflexi, Firmicutes*, and *Rokubacteria* were found in all the tested sites, although at different relative abundance. On the opposite, in ILVO, *Entotheonellaeota, Patescibacteria, Elusimicrobia, Latescibacteria*, and *Verrucomicrobia* phyla were absent. *Chlamydiae* phyla was the unique phyla not found in CREA site, while *Nanoarchaeaeota, Halanaerobiaeota, Zixibacteria, Omnitrophicaeota, Spirochaetes, Hydrogenedentes, Deinococcus-Thermus*, and *Epsilonbacteraeota* were found only at ILVO experimental site, and *Dependentiae* and *Fibrobacteres* only at AU (Figure 7).

In relation to fungi phyla, *Basidiomycota, Ascomycota, Mucoromycota, Mortierellomycota*, and *Chytridiomycota* were ubiquitarian in all the tested systems, while *Kickxellomycota* were found only in CREA experimental site, being instead absent *Rozellomycota* and *Aphelidiomycota* phyla, which were recorded in ILVO and AU systems. In AU system, *Glomeromycota* very low relative abundance was not accounted under cabbage and beetroot MC (0.00071% under cabbage MC and 0.00443% under beetroot MC), although beetroot showed a certain mycorrhizal colonization intensity (<6%) (Figure 7).

We also investigated the effects of crop and CS on potential pathogens population through NMDS and PERMANOVA at CREA and ILVO sites. The NMDS did not yield a clear relationship between crop and CS on pathogens relative abundance and structure in both sites (Supplementary material 4). Table 3 shows the results of the PERMANOVA carried out on the relative abundance of the selected fungal pathogens population. At CREA the model explained about 38% of the total variability. Crop (*R*^2^ = 0.24; *p* ≤ 0.05) showed a significant effect on pathogens population. Similarly crop (*R*^2^ = 0.11; *p* ≤ 0.001) and block (*R*^2^ = 0.06; *p* ≤ 0.01) showed a significant effect on pathogens community at ILVO site, where the model explained about 22% of the whole variability.

**Table 3:** Results of the PERMANOVA carried out on the CREA and ILVO dataset on selected fungal pathogens.

	**CREA**		**ILVO**	
	**R^2^**	**Pr(>F)**	**R^2^**	**Pr(>F)**
Cropping system	0.07	n.s.	0.02	n.s.
Crop	0.24	^*^	0.11	^***^
Block	0.04	n.s.	0.06	^**^
Cropping system × crop	0.03	n.s.	0.03	n.s.

^*^, ^**^, and ^***^ significant at p ≤ 0.05, p ≤ 0.01, and p ≤ 0.001, respectively; n.s., not significant.

**Figure 8:**
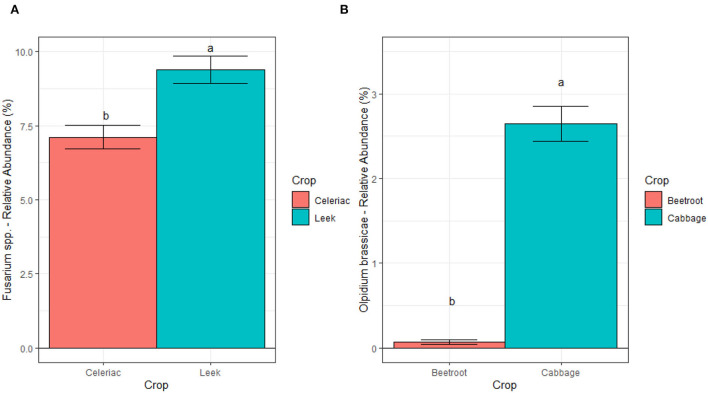
Relative abundances of *Fusarium* spp. at ILVO site **(A)** and of *Olpidium Brassicae* at AU site **(B)**, averaged across cropping systems and blocks. Bars denote standard errors of the mean. Different letters are significantly different at *p* < 0.05 (Tukey test).

We further studied the effect of crop and CS on *Fusarium* spp. at ILVO and CREA sites, and on *Olpidium brassicae* at AU through regression models. At ILVO, the analysis highlighted a significant effect of crop, with the higher relative abundance of *Fusarium spp*. under leek as compared to celeriac (Figure 8).

**Figure 9:**
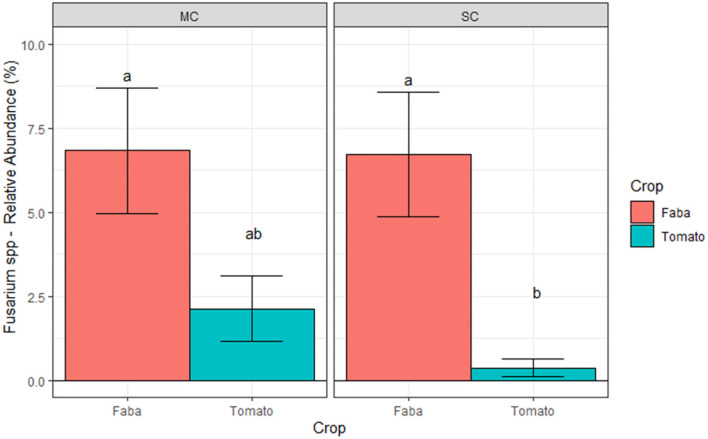
Relative abundances of *Fusarium spp*. at CREA site averaged across blocks. Bars denote standard errors of the mean. Different letters are significantly different at *p* < 0.05 (Tukey test). MC, Monocropping; SC, Strip cropping.

Similarly, the comparison across the different crop monocultures at AU showed a strong crop effect on *Olpidium brassicae* relative abundance. Here, the relative abundance of this plant pathogen was about eight times higher under cabbage compared to beetroot (Figure 8). A different scenario was observed at CREA site, where we found a significant interaction between crop and CS (Figure 9). We observed a lower relative abundance of *Fusarium spp*. in tomato rhizosphere under SC compared to MC, when the taxonomic attribution of the AVS identified *F. oxysporum, F. solani, F. nematophilum, F. proliferatum*, and 2 ASV of unidentified *Fusarium*. Nevertheless, the *post hoc* test did not highlight significant differences in relative abundance of this genus, potentially pathogenic, on tomato under the two CS.

**Figure 10:**
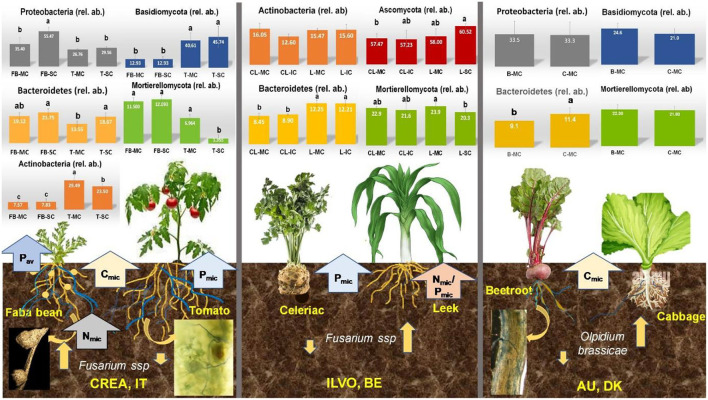
Synthesis of main relevant results obtained at CREA (IT), ILVO (BE), and AU (DK) experimental sites on belowground functional diversity. Bars denote standard errors of the mean. Different letters are significantly different at *p* < 0.05 (Tukey test). MC, monocropping; SC, strip-cropping; IC, intercropping.

In Figure 10, a schematic representation of the main results obtained at CREA, ILVO, and AU experimental sites are described.

A linkage among the increase in relative abundance *of Proteobacteria*, the decrease in *Actinobacteria*, and the highest soil P_av_ was observed under SC faba bean at CREA, not recorded at ILVO celeriac–leak. At the same time, an increase in *Bacterioidetes* was found under both crops under SC at CREA, and under MC and IC leek at ILVO. On the opposite, a decrease of *Mortierellomycota* relative abundance was observed in tomato SC at CREA and leek IC at ILVO site, where P_mic_ was the highest. Interestingly, when C_mic_ was increased and P_mic_ was the highest, the mycorrhizal colonization of the crop increased, as at CREA and at AU.

## Discussion

We hypothesized that, in studying multi-cropping vegetable systems, the plant diversity is able to shape soil microbial community supplying several ecosystem services, such as improved soil C-N-P cycles, increased plant mycorrhization, and reduced fungi soil-borne diseases. Actually, the coexistence in the field of plants with different belowground traits (Schmid et al., 2021) can lead to: i) a higher microbial community diversity in the rhizosphere, due to an additive interaction effect between the microbiota associated with both crops (complementary relationship among microbial species); ii) a lower microbial community diversity at the belowground, due to the migration of specific bacteria or fungi groups toward the rhizosphere soil, associated with the most “affine” crop (selection among microbial species). To verify the hypotheses, a multifunctional approach was applied.

### Soil parameters and microbial stoichiometry

Soil organic carbon (SOC) and nutrient pools affect soil microbial stoichiometry by influencing bacteria or fungi dominance. Soil disturbance usually lowers TOC, organic N, and increases P_av_ content. This build up the diversity of low C_*mic*_:P_*mic*_ fast-growing bacteria, which require high P_av_ to support their high growth rate (Delgado-Baquerizo et al., 2017). In natural or less disturbed soils, TOC and organic N increased, reducing fast-growing bacteria diversity in favor of fungi communities' dominance, particularly mycorrhizal fungi, which exploit soil P sink by the development of hyphal network (Schnepf et al., 2008; Chen et al., 2016). The soil pH is another key soil indicator affecting soil microbiota, working mainly on the relative abundance of principal decomposers groups of fungi and bacteria. A decrease in fungi/bacterial growth ratio was observed going from pH 5.0 to 8.0 (Rousk et al., 2009). In our sites, overall pH was inversely correlated with bulk density, HWC, and HWP, and positively correlated with C_mic_/TOC and P_mic_, thereby influencing soil microbial biomass amount and quality (Table 2).

At CREA site, a shift toward bacteria community was found under faba bean SC (C_mic_:N_mic_ >>5, >N_mic_:P_mic_, Rousk et al., 2009), while a net dominance of fungi communities under tomato SC was observed (C_mic_:N_mic_ >5, < N_mic_:P_mic_ ratio, Zhang and Elser, 2017) (Table 2). Since soil nutrient stoichiometry is one of the main predictors of microbial biomass composition (Strickland and Rousk, 2010; Delgado-Baquerizo et al., 2017), we argue that the lowest N_mic_ under faba bean SC was probably driven by the low availability of soil mineral N (7.3 mg kg^−1^) at harvest under N-fixing legume crop, in absence of N-fertilization. Conversely, the highest soil mineral N (10.8 mg kg^−1^) in tomato MC at harvest is the result of N-rich legume residue degradation by soil microbial communities, combined with the N input from fertilizer.

At ILVO, celeriac–leek IC induced a shift of soil microbes toward fungal communities. The very low N_mic_:P_mic_ ratio suggests the dominance of saprotrophic groups in IC (Zhang and Elser, 2017). In contrast, beetroot–white cabbage IC in AU site apparently did not affect bacteria/fungi dominance, being microbial stoichiometry not changed by multi-cropping. However, the increase of C_mic_ and C_mic_/TOC recorded under IC, compared to MC (Table 2), testifies to the ecosystem service provided by crop diversification on improving soil C stock by microbial biomass.

### Crop root mycorrhization

Considering the ecosystem service provided by mycorrhizal fungi in supporting plant productivity and quality in organic systems (Gianinazzi and Gollotte, 2010; Verbruggen et al., 2010), we assert that multi-cropping can favor beneficial symbioses. As an example, the combination of different plants with supporting arbuscular mycorrhizal trait coexisting in the field supports the development of mycorrhizal hyphal network, thus boosting mycorrhizal colonization of the whole agroecosystem (Simard and Durall, 2004; Trinchera et al., 2016). We found evidence of the beneficial role played by crop diversification on root mycorrhization at the CREA site, where faba bean and tomato were more colonized by mycorrhizal fungi in SC than in MC (Figure 4A).

In AU site, we instead expected that the presence of intercropped cabbage would have completely contrasted root mycorrhizal colonization in field (Lebeis, 2015): in fact, *Brassicacea*e plants activate defense mechanisms based on glucosinolates hydrolysis (Lüthy and Matile, 1984), which generate a bacteria-dominated microbiome and no mycorrhiza harboring (Rumberger and Marschner, 2003). Although *Glomeromycota* phylum was not relevant in terms of relative abundance (Figure 8), the coexistence of rhizosphere microbial communities under cabbage–beetroot IC apparently reduced the strong inhibiting effect played by cabbage on mycorrhizal fungi population at AU site. The presence of weeds species with supporting arbuscular mycorrhizal trait, such as *Capsella bursa-pastoris* L., *Senecio vulgaris* L., *Spergula arvensis* L., and *Plantago maior* L. (results not shown), recorded in beetroot IC only, evidently promoted the beetroot mycorrhization (Figure 4B), overruling the inhibiting effect played by the nonmycorrhizal cabbage (Hajiboland et al., 2020; Trinchera et al., 2021). This finding underpinned the hypothesis that different species of plant in the field play a selection of rhizosphere microbiome according to the plant functional traits: here, by promoting the migration of beneficial fungi population toward the beetroot (Trinchera et al., 2016).

### Rhizosphere bacterial/fungal diversity

As far as the microbial functional diversity is concerned, multi-cropping did not translate into a highest bacteria/fungi community diversity, while a fungi phyla selection took place under SC at CREA site, mainly driven by legume species (Figure 5). In fact, faba bean showed a predominant role in shaping bacteria/fungi community at the belowground of both the companion crops: *Proteobacteria*, which also *Rhizobium* genus belong to, dominated the belowground diversity under the legume, being mainly α*-*, but also β*-* and γ*-Proteobacteria* associated with legume nodulation (Benhizia et al., 2004; Mus et al., 2016) (Figure 6). The presence of *Rhizobia* in soil as N-fixing bacteria largely depends on the secretions of the legume roots, which include both high and low molecular weight compounds acting as cues in plant–microbe signaling and recognition (Biate et al., 2014). In the late spring season, the presence of tomato roots growing close to faba bean ones in SC boosted the *Proteobacteria* accumulation around the faba bean roots. This is a first evidence of these bacteria selection induced by the legume root exudates, which promoted their migration toward legume roots and far from tomato ones, being this last one a *Rhizobia* nonhost plant. *Acidobacteria*, typically aerobes phyla, prefer low pH soils and operate by decomposing organic substrates and storing soil C as microbial biomass: they represent a core bacterial component among rhizobacterial assemblages, comprising 10.7% of the total observed operational taxonomic units (Na et al., 2019). They were well represented under faba bean MC, where the association with N-fixing bacteria was evidently favored but decreased rapidly in tomato SC rhizosphere soil, where the soil pH was the highest. *Actinobacteria* phyla instead were mainly associated with tomato, although a decrease was detected under tomato SC compared to MC. These phyla are mainly involved in fast degradation of low biodegradable organic compounds, such as hydrocarbons, lignin, and humus. Their decrease under tomato SC again indicated the selection effect played by legume crop, being these phyla underrepresented under faba bean. *Bacteroidetes* were well represented in CREA site regardless of CS. Generally, the impact of plant domestication process on rhizobacterial community composition leads to a decrease in *Bacteroidetes* relative abundance, while increasing *Actinobacteria* and *Proteobacteria* one (Pérez-Jaramillo et al., 2018). From an ecological point of view, soil *Bacteroidetes* thrive because of their ability to secrete diverse arrays of carbohydrate-active enzymes that target the highly varied glycans in soil, which carry out detritus decomposition (Larsbrink and McKee, 2020). The observed results evidenced the ability of crop diversification to modulate bacterial community composition in favor of those microbial groups generally reduced in agricultural, disturbed soil (Wolińska et al., 2017), due to the repeated mono-cultivation of highly domesticated varieties.

In relation to fungal community, *Ascomycota* were the predominant phyla under legume crop, while both *Ascomycota* and *Basidiomycota* were well-represented under tomato. The function of *Basidiomycota* is relevant in terms of organic matter stabilization in soil (microbial coefficient higher under tomato compared to faba bean). We stress that these fungi phyla play a key role in system ecology, due to their involvement in C cycling in temperate systems, as wood decomposers and ectomycorrhizal symbionts. For example, they form underground resource-sharing networks which support plant biodiversity in forest ecosystems (Taylor et al., 2015). Their lower relative abundance under faba bean at CREA highlights that the repeated introduction of legume species in crop rotations or in multi-cropping systems may reduce the relative abundance of *Basidiomycota* in bulk soil in the long term. This should be taken into consideration when designing organic multi-cropping systems, where SOM is subjected to high mineralization, such as those of Mediterranean region. Interestingly, *Mortierellomycota* showed the opposite behavior. They are non-saprotroph fungi phyla, living in soil on decaying leaves and other organic materials. Again, the plant made the difference, their relative abundance being highest under faba bean, independently from CS, and lowest under tomato SC. *Mortierellomycota*, and particularly of *Mortierella* genus (the most abundant genus in CREA system, see Supplementary material 3), promote plant growth across different types of crops, including herbaceous crops, so that to be considered a potential bioindicator and biocontrol agent for crop production and soil health (Zhang K. et al., 2020). *Mortierella* genus was associated with tomato rhizosphere at CREA site. The observed decrease in tomato SC was probably due to the proximity of tomato–faba bean roots and the presence of flattened legume residues as green mulch, which mostly favored the association of saprophyte, active decomposer fungi population (*Ascomycota*) with fungi groups involved in organic matter resynthesis (*Basidiomycota*). At last, we found that the increased mycorrhizal colonization observed under tomato SC did not correspond to an increase of *Glomeromycota* relative abundance in rhizosphere soil.

The decrease in *Actinobacteria* under celeriac IC at ILVO site, counteracted by the increase in relative abundance of *Proteobacteria* (the overrepresented bacteria phyla), again confirms the selection effect played by two different crops contemporary grown in field. *Bacteroidetes* relative abundance was the highest under leek: it was already found that *Bacteroidetes* increased as a response of crop diversification in a banana–leek rotation, compared to banana MC (Ouyang et al., 2011). Consequently, the fluctuation of *Bacteroidetes* relative abundance appeared a suitable microbial indicator to evaluate the effect of crop diversification on soil bacteria diversity. Concerning fungi, a transition toward *Ascomycota*-dominated community was observed in celeriac IC, indicating that fungal community shifted from slow-growing oligotrophic fungi groups to fast-growing copiotrophic fungi groups (Yang et al., 2017). Again, the decrease of *Mortierellomycota* in both IC celeriac and leek suggests that the shared roots between different companion crops exert a negative effect on relative abundance of this phyla. This result was already observed in other intercropped systems, where a decrease in *Mortierella* genus was detected. However, the reason for this reduction was not fully clear and calls for further investigations (Sen and Fengzhi, 2018).

In AU site, *Proteobacteria* and *Actinobacteria* dominated both cabbage–beetroot MC bacteria community, confirming that again a certain impact of domestication on rhizobacterial diversity took place. Unexpectedly, the crops did not change bacterial community composition, but a significant increase in *Bacteroidetes* relative abundance was observed under cabbage. In relation to fungi community composition, *Ascomycota*, the most representative phyla, *Basidiomycota*, and *Mortierellomycota* were predominant, again regardless of the companion crops. The high relative abundance of *Mortierellomycota* found in AU site is an evidence of good soil health, since *Mortierella*, the most represented genus among *Mortierellomycota*, can synthetize the arachidonic acid, recognized as an elicitor of phytoalexins in plants for suppressing plant disease (Eroshin et al., 1996; Tagawa et al., 2010). The absence of *Olpidiaceae* phyla in beetroot rhizosphere, instead detected in the cabbage one, has an important agroecological implication. Crop diversification has been often reported to be a viable strategy to decrease fungal pathogens accumulation with positive feedback on crop productivity (Maron et al., 2011). The mechanisms behind this effect include: (i) the exudation of allopathic compounds (e.g. Hao et al., 2010); (ii) reductions in the relative abundance of pathogens due to an increase diversity and physical occupation of pathogens niches (Mitter et al., 2013); (iii) reductions in pathogens host plant species due to higher crop diversity (He et al., 2019); and (iv) positive effects on antagonistic microbial communities (Latz et al., 2012). In this study, when we aggregated the data on fungal pathogens, we did not find a significant effect of the CS on pathogens communities at CREA and ILVO sites (Table 3). The relative abundance of specific *genera*, which also comprise pathogens (namely, *Fusarium* spp. and, particularly, *Olpidium brassica*) (Bolwerk et al., 2005), which are known to be cause of serious plant diseases, was significantly affected by crops (Figures 8, 9). From a field perspective, this result supports the idea that combining a nonhost with a host plant can limit the relative abundance of pathogens in long term, with positive effect on crop productivity. Only at CREA site, we also found an interaction effect of crop diversification in the rhizosphere, being *Fusarium* spp. less abundant under tomato when grown in SC compared to MC. Although the *post-hoc test* did not significantly discriminate across the different CS, this trend suggests a possible positive effect of multi-cropping system in diminishing *Fusarium spp* in tomato cultivation. The lack of a strong CS effect on the selected fungal pathogens was probably due to the sampling protocol: we indeed sampled the rhizosphere soils as a mean to investigate whether roots interactions under IC would have affected fungal pathogens populations. Probably, a bulk soil sampling could have yielded more solid information on the pathogens population under IC as compared to MC.

In conclusions, IC and SC may increase soil microbial biomass, N and P nutrient availability, thus shaping the microbial community toward predominance of bacteria or fungi community, in function of selected companion crops and pedoclimatic conditions. Multi-cropping does not increase the overall bacteria and fungi diversity, while we observed a crop selection effect on rhizosphere microbiota, rather than a complementary, additive effect among microbial species. In multi-cropping systems, increased *Bacteroidetes* and reduced *Mortierellomycota* relative abundance in rhizosphere soil suggest they can be considered as sensitive ecological indicators of improved agro-system functionality induced by plant diversity: *Bacterioidetes*, being able to testify the introduction of low-impact agricultural practices, and *Mortierellomycota* as undirect indicator of the reduced pressure made by pathogens. Multi-cropping favored the spontaneous mycorrhizal symbiosis between companion crops, leading to a corresponding increase of SOC accumulation in microbial biomass. Where companion crops were duly selected, multi-cropping also reduced the relative abundance of soil pathogens, with a potential positive effect on crop productivity in the long term. However, further studies are requested to understand the role played by fungal hyphae on SOC accumulation and by crop mycorrhization in reducing fungal pathogen in multi-cropping systems.

## Data availability statement

Used bacteria and fungi primers and bacteria/fungi counts presented in this study are deposited in DRYAD repository “Supplementary materials 2, 3_Bac_Fungi primers and Bac_Fungi counts_Article: “Can multi-cropping affect soil microbial stoichiometry and functional diversity, decreasing potential soil-borne pathogens? A study on European organic vegetable cropping systems” _Frontiers in Plant Science”. Accession number: https://doi.org/10.5061/dryad.kh1893296.

## Author contributions

AT: first authorship, conceptualization, dataset elaboration, evaluation of root mycorrhization, results interpretation, and final revision. MM: DNA extraction, identification of bacteria/fungi rhizosphere populations from NGS dataset, and support to first authorship on Rhizosphere bacteria/fungi diversity section. DW statistical analysis and support to first authorship on paper revision. SO: DNA extraction and identification of bacteria/fungi rhizosphere populations from NGS data. JD: support on protocols for rhizosphere sampling and NGS analysis, NGS data interpretation, and paper revision. SS: stoichiometry determinations, sampling and evaluation of mycorrhizal colonization dataset, and paper revision. SD: sampling and evaluation of mycorrhizal colonization dataset and paper revision. JB: setting of experimental design and soil parameters determination. PK: determination of soil parameters and paper revision. HK: setting of experimental design and final paper revision. LL and TS: setting of experimental design and paper revision. GC: setting of experimental design. KW: senior authorship, conceptualization, and final revision. All authors contributed to the article and approved the submitted version.

## Funding

This research was approved by Core Organic Cofund 2016-2017 (grant number n. 1954) and funded by Italian Ministero dell'Istruzione, dell'Università e della Ricerca (MIUR, D.D. n. 313 of 06/03/2019, March 2018-November 2021). The APC was funded by Italian Ministero delle politiche agricole alimentari e forestali (Mipaaf) within the project METinBIO (Admissibility of fertilizers for organic production, grant number Mipaaf n.76831 of 31/10/2018 (2019-2023)).

## Conflict of interest

The authors declare that the research was conducted in the absence of any commercial or financial relationships that could be construed as a potential conflict of interest.

## Publisher's note

All claims expressed in this article are solely those of the authors and do not necessarily represent those of their affiliated organizations, or those of the publisher, the editors and the reviewers. Any product that may be evaluated in this article, or claim that may be made by its manufacturer, is not guaranteed or endorsed by the publisher.
