# Supplementary material for: Can multi-cropping affect soil microbial stoichiometry and functional diversity, decreasing potential soil-borne pathogens? A study on European organic vegetable cropping systems
Source: Front Plant Sci. 2022 Sep 27;13:952910. doi: 10.3389/fpls.2022.952910 (PMC9552534; doi:10.3389/fpls.2022.952910)

Non metric multidimensional scaling on pathogens data for the ILVO site. IC: Intercropping; MC= monocropping


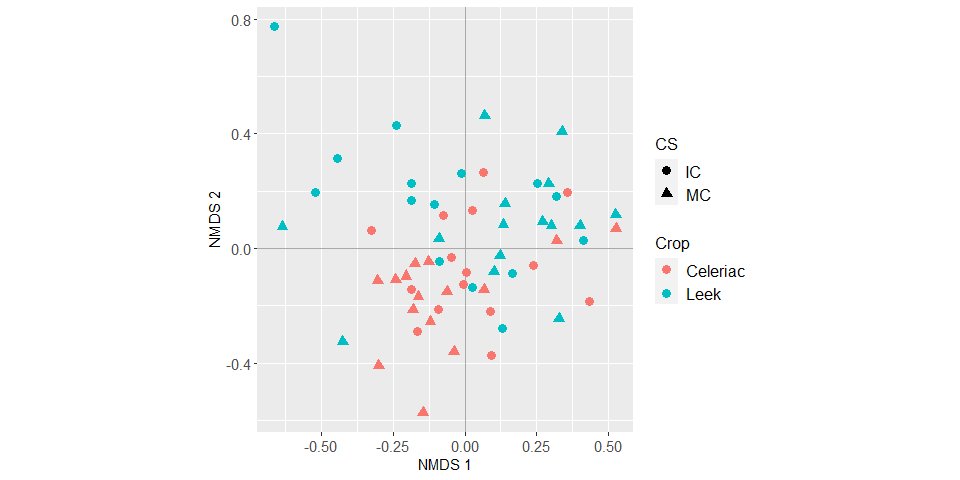


1. Non metric multidimensional scaling on pathogens data for the CREA site. SC: stripcropping; MC= monocropping


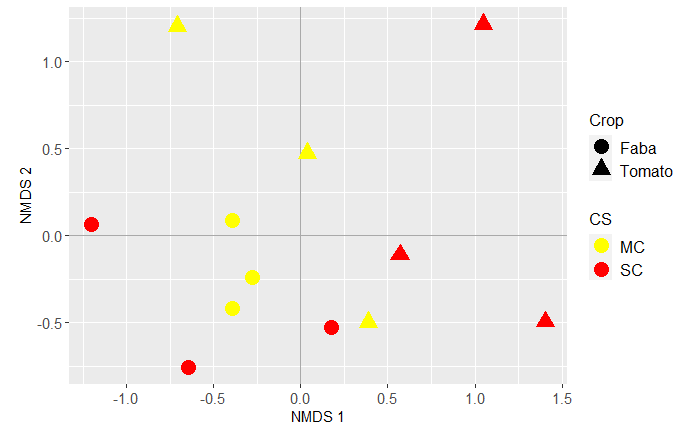

Supplement: Supplementary file 1 [file Data_Sheet_1.zip › Supplementary Material/Supplementary Material 4.docx]
