# Supplementary figures and images for: Can multi-cropping affect soil microbial stoichiometry and functional diversity, decreasing potential soil-borne pathogens? A study on European organic vegetable cropping systems
Source: Front Plant Sci. 2022 Sep 27;13:952910. doi: 10.3389/fpls.2022.952910 (PMC9552534; doi:10.3389/fpls.2022.952910)

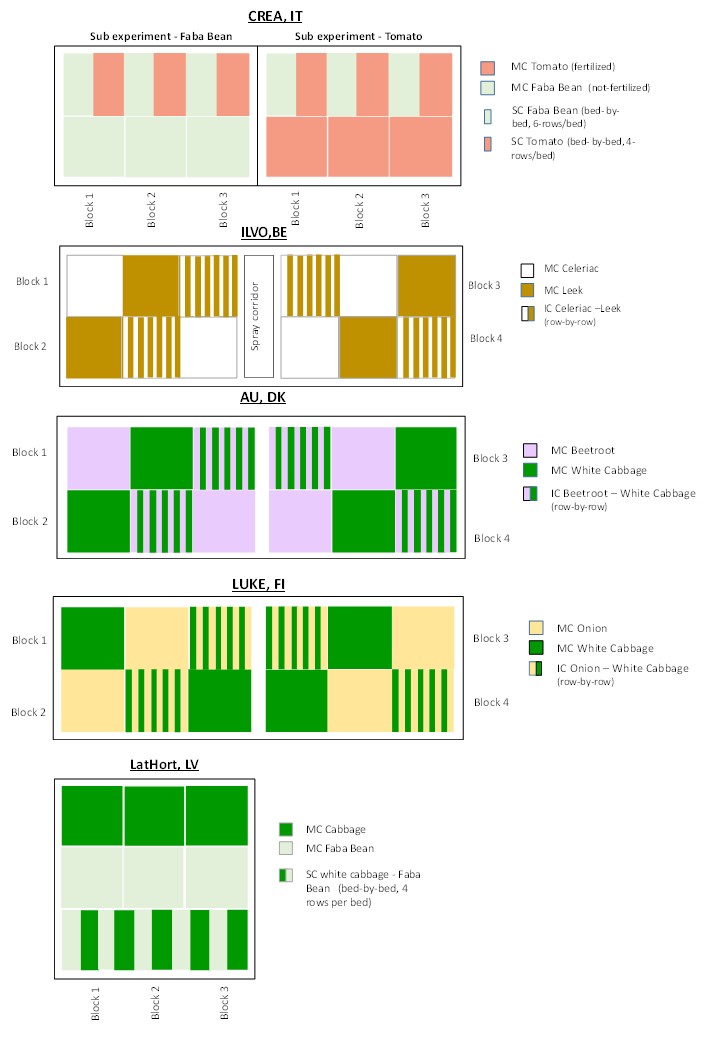

Supplement: Supplementary file 1 [file Data_Sheet_1.zip › Supplementary Material/Supplementary Material 1.JPEG]
